# Current News

**Published:** 1890-01

**Authors:** 


### Current News.

To induce an alkaline condition of the oral secretions during pregnancy, Dr. Jno. H. Coyle prescribes fifteen grains bicarbonate of potassa, in cinnamon-water, fifteen minutes after each meal.

---

Dr. R. B. Adair syringes the pocket of an alveolar abscess with equal parts of peroxide of hydrogen and bichloride of mercury one to five hundred.

---

Dr. Wardlaw thinks the influence of sex positive as a predisposing cause of caries, the excessive liability in the case of pregnant females being explained upon the principle of the vitiated condition of the fluids of the mouth consequent upon systemic disorder, their corrosive properties being thus increased.

---

For starting a filling in a large cavity, Dr. W. C. Browne places in the bottom a pellet touched on the under-side with damar dissolved in chloroform.

---

Dr. H. S. Colding thinks that the best way to make a dentist is to select a boy with good mechanical abilities, and put him to work at the machinist's trade for two or three years; then give him the spring and summer course in the infirmary of a first-class dental college. He thinks this a better preparation for the college course proper than years under a preceptor.

---

Antiseptic mouth-wash, Dr. S. A. White's: four drachms carbolic acid crystals; three ounces glycerine; three ounces water. Use with soft tooth-brush.

---

Keep on hand a supply of Belding Bros. embroidery silk on small spools; teach your patients how to use it, and give the patient a spool, exacting the promise of frequent use. The cost, one cent a spool, is nothing compared with the good you will do.

B. H. Catching.

A COMPLETE, separate, cross-topical index is now in preparation. Do not have Volume X. bound until you receive it.

The topical index for Volume X. will be bound separately this and succeeding years.

Do not make the mistake of having the topical index for Volume IX.—contained in the January number of Volume X.—bound in the latter volume, but wait till you receive the topical index for Volume X., which will be ready in a few days.

The topical index for Volume IX. has been so popular that other and rival journals have adopted our idea; but to the INTERNATIONAL belongs the credit of introducing an exhaustive annual topical index.

---

### CHICAGO AND VICINITY.

DR. ALLPORT treats hyperæmia of the pulp due to thermal changes, after inserting large gold fillings, by covering the filling with oxyphosphate of zinc cement, and renewing it as occasion requires until such time as normal action is re-established.

---

THE Chicago Dental Society is making preparations for its annual banquet. We hear that strict prohibition is to be enforced, that nothing stronger than apollinaris and cigars will be permitted.

---

RECENT news in regard to the health of our friend, Professor Truman W. Brophy, is not very encouraging, but we hope to be able to give a more favorable report later.

---

DR. FERNANDEZ, of Chicago, has a neat little wrench for use with the Brophy band matrix. It is constructed with a flexible shaft and can be used at any angle. The shank of a watch-key is soldered to a short piece of steel coil spring, and the opposite end of the spring to a straight shaft of any desired length. It can be made in a few minutes and is very useful.

---

LA GRIPPE has reached Chicago, and many of her citizens are in its *grip* just at present. So far it has not assumed a serious form, and, no doubt, out of respect for our record as the most healthy city in the union, it will touch us mildly.

